# Freedom from Recurrence across Age in Non-Melanoma Skin Cancer Treated with Image-Guided Superficial Radiation Therapy

**DOI:** 10.3390/geriatrics9050114

**Published:** 2024-09-05

**Authors:** Aaron S. Farberg, Randy V. Heysek, Robert Haber, Rania Agha, Kevin M. Crawford, Ji Xinge, Jeffrey Blake Stricker

**Affiliations:** 1 Bare Dermatology, Dallas, TX 75235, USA; aaron.farberg@gmail.com; 2 University of North Texas Health Science Center, University of North Texas, Fort Worth, TX 76107, USA; 3 Central Florida Cancer Institute, Davenport, FL 33837, USA; heysek@cfcancerinst.com; 4 Department of Dermatology, Case Western Reserve University School of Medicine, Cleveland, OH 44106, USA; bobhaber2@gmail.com; 5 Department of Dermatology, The University of Illinois at Chicago, Chicago, IL 60607, USA; agharaniamd@gmail.com; 6 Jesse Brown VA Medical Center, Chicago, IL 60612, USA; 7 Department of Medicine, Rosalind Franklin University of Medicine and Science, North Chicago, IL 60064, USA; 8 Department of Dermatology, Marian University College of Osteopathic Medicine, Indianapolis, IN 46222, USA; 9 Department of Dermatology, Indiana University School of Medicine, Indianapolis, IN 46202, USA; 10 Independent Researcher, Champaign, IL 61822, USA; jixinge1119@gmail.com; 11 Dermatology Specialists of Alabama, Dothan, AL 36303, USA; 12 Alabama College of Osteopathic Medicine, Dothan, AL 36303, USA; 13 Southeast Health Internal Medicine Residency, Dothan, AL 36301, USA

**Keywords:** non-melanoma skin cancer, image-guided superficial radiation therapy, freedom from recurrence, age, sex, stage

## Abstract

Non-melanoma skin cancers (NMSCs) are a significant cause of morbidity and mortality; their incidence is increasing most in older patients. NMSCs have traditionally been treated with surgical excision, curettage, Mohs micrographic surgery (MMS), and superficial radiotherapy (SRT). Image-guided SRT (IGSRT) is a treatment option for poor surgical candidates or patients with low- or high-risk, early-stage NMSC who prefer to avoid surgery. This large retrospective cohort study compared 2-, 4-, and 6-year freedom from recurrence in biopsy-proven NMSC lesions treated with IGSRT (*n* = 20,069 lesions) between patients aged < 65 years (*n* = 3158 lesions) and ≥65 years (*n* = 16,911 lesions). Overall freedom from recurrence rates were 99.68% at 2 years, 99.57% at 4 years, and 99.57% at 6 years. Rates did not differ significantly by age (*p* = 0.8) nor by sex among the two age groups (*p* > 0.9). There was a significant difference in recurrence among older patients when analyzed by stage (*p* = 0.032), but no difference by stage in younger patients (*p* = 0.7). For early-stage NMSCs, IGSRT is a clinically equivalent alternative to MMS and statistically significant in superiority to non-image-guided SRT. This study demonstrates that there is no significant effect of age on 2-, 4-, or 6-year freedom from recurrence in patients with IGSRT-treated NMSC.

## 1. Introduction

Non-melanoma skin cancers (NMSCs) are tumors that include basal cell carcinomas (BCCs, constituting 70% of NMSCs), squamous cell carcinomas (SCCs, constituting 25% of NMSCs), and squamous carcinoma in situ (SCCIS) [1,2]. NMSCs constitute approximately one-third of malignancies diagnosed globally [3] and are the most prevalent cancers in the United States [4], with an incidence for basal cell carcinoma of 525 per 100,000 persons and for squamous cell carcinoma of 262 per 100,000 persons in 2019 [5]. Global incidence of and disease burden attributable to NMSCs are expected to increase by at least 1.5 times from 2020 to 2044 [6]. This increase in incidence is attributable to a variety of factors, such as longer lifespans, ozone depletion, and increased exposure to ultraviolet light [2].

The incidence of BCC is increasing most precipitously in lighter-skinned men aged ≥ 65 years [7], with rates particularly high in patients with a history of ionizing radiation exposure, a history of chronic arsenic ingestion, immunosuppression, or family history [8]. Late detection and diagnosis are common in older patients, particularly in the context of cognitive and functional impairment, mood disorders, low socioeconomic status, and insufficient social support [9]. This delay in detection can result in tumors that are extensive, have caused significant destruction of local tissue, and may not be amenable to surgical treatment [10]. Older patients with NMSC are also more likely to have multiple comorbidities and contraindications for general anesthesia or surgery [11] and may be more affected by the impact of NMSC on their health-related quality of life, such as difficulties associated with infection, inflammation, function loss, tumor-related skin disruption, aesthetic effects, or exudation [12]. Unfortunately, older patients are underrepresented in clinical trials [13] and treatment decisions are therefore often made on the basis of case discussions, clinical experience, published recommendations, and literature reviews [14,15,16].

Current National Comprehensive Cancer Network (NCCN) guidelines for the treatment of localized but low- and high-risk BCCs and SCCs include surgical excision or Mohs micrographic surgery (MMS), with definitive radiation therapy recommended for patients who are poor surgical candidates or who prefer a nonsurgical approach in cosmetically sensitive areas, as the primary treatment goal is complete removal of the tumor and the maximal preservation of function and cosmesis. All treatment decisions should be customized to account for the individual case and for the patient’s preference [17,18]. MMS is a precise, tissue-sparing method of removing skin cancer that allows for microscopic control of the whole tumor margin while conserving as much healthy tissue as possible [19]. In older patients, it is most commonly used when patients have high functional status, high-risk tumors, and tumors located in the face [20]. Patients aged ≥ 80 years undergoing MMS have been reported to have tumors with deeper invasion and require a higher number of Mohs surgery stages, which results in larger defects and more operative time [21]. MMS has been reported variously to have an overall complication rate of 7% in patients aged ≥ 80 years [21] and 1.78% in patients aged ≥ 85 years [22], with complications including infection, wound dehiscence, hematoma, hemorrhage, flap necrosis, and graft necrosis [22].

MMS has replaced superficial radiation therapy (SRT) as the preferred treatment modality [23]. SRT is a form of radiation that uses low-energy kilovoltage photons to confine treatment to the skin. SRT dosing schedules usually vary from 5 Gy/fraction ×7 fractions (35 Gy total) to 2 Gy/fraction × 30 fractions (60 Gy total) or more depending on patient age, lesion size, and lesion location [24]. SRT may be appropriate for older patients with BCC or SCC lesions in the lower extremities when they may otherwise suffer complications or prolonged healing after surgery. A retrospective review of 38 cases of BCCs and 113 of SCCs and a mean patient age of 82.5 years found an overall SRT success rate of 97.4% [25].

A newer treatment modality the United States Food and Drug Administration (FDA) cleared in 2015 for NMSC, image-guided superficial radiation therapy (IGSRT), uses an integrated high-resolution dermal ultrasound technology to improve lesion visualization, which allows for more precise radiation targeting due to a more accurate assessment of the tumor depth, width, and breadth, allowing clinicians to provide adaptive radiation treatment planning. In IGSRT, an ultrasound set to a frequency of 22 MHz, which is the optimal frequency for evaluating a skin layer with a depth of 0–6 mm, is used to determine the extent of the lesion beyond clinical visibility [26]. IGSRT has demonstrated a 99.3% rate of local tumor control in 2917 NMSC lesions [26]. It has also demonstrated a 99.7% absolute lesion control rate in 1899 lesions with an average of 7.5 weeks of treatment, a stable control rate of 99.6% with follow-up of >12 months, and local control rate of 99.41% at the maximum follow-up of 63.6 months [27]. Several retrospective cohort studies have also shown that IGSRT for early stage NMSCs is a clinically equivalent alternative to MMS and statistically significant in superiority to traditional non-image-guided SRT and other radiation therapy technologies. In comparison with traditional SRT, IGSRT has demonstrated statistically superior 2-year recurrence rates (0.7% overall, 1.1% for BCCs, 0.8% for SCCs, and 0.0% for SCCIS) in a retrospective cohort study of 2880 lesions. A meta-analysis of two studies evaluating IGSRT and four studies evaluating traditional SRT further found that local control of early-stage high- and low-risk NMSCs with IGSRT was statistically superior to traditional SRT overall and in all subtypes when stratifying by histology [28,29]. However, there is now a need for larger cohort studies to allow for the evaluation of recurrence after IGSRT by patient and disease characteristics, such as patient age, sex, and stage.

The objective of this large retrospective cohort study was therefore to determine the effect of patient age on 2- and 5-year freedom from recurrence rates in patients with NMSCs treated with IGSRT, comparing patients aged ≥ 65 years vs. those aged < 65 years, with further analyses based on sex and stage.

## 2. Materials and Methods

### 2.1. IGSRT Treatment Methodology and Energy/Dose Selection Process and Tumor Configuration and Depth Determination

Treatment methodology has been described in detail in [26,27] and follows a general guideline (the Ladd–Yu protocol) [26] for treatment dose, energy, fractionation, and therapeutic biologic effect represented by time–dose–fractionation (TDF) calculations. A standardized protocol with a total of ~20 fractions using single energy or a sequential combination of 50 kVp, 70 kVp, or 100 kVp energy X-ray treatment is generally delivered 2–4 times per week with pre-treatment daily high-resolution dermal ultrasound (HRDUS) prior to “beam-on” to assess/confirm tumor configuration/location and detect changes which may indicate a prescription change is necessary with adaptive radiation treatment planning. HRDUS is also performed during initial simulation for treatment planning purposes as well as at follow-up evaluations after treatment course completion to evaluate response.

HRDUS uses a non-invasive 20–22 MHz ultrasound with Doppler component probe that is intrinsic to the IGSRT unit (Sensus SRT-100 Vision) which allows for the visualization of 0–10 mm into the skin structure, including a visualization of the epithelium, papillary layer, and sometimes down to the reticular layer depending on anatomic location and skin thickness. This high-resolution/high-frequency ultrasound allows for a clear visualization of normal skin anatomy and the disrupting tumor, which occupies a black space without Doppler color speckles, and allows for precise visualization, measurement, and the capture of the exact depth of penetration, allowing the clinician to perform adaptive radiation therapy planning during a course of care. The width and configuration of the tumor can also be easily discerned with HRDUS and is integral to localization and treatment planning, reducing the risk of anatomical miss and misadministration.

### 2.2. Data Collection

Data collection followed a similar process as described in published studies [26,27]. IGSRT treatment records of 19,935 NMSC lesions treated at 28 institutions across 12 states from 2016 through to 2023 were retrospectively gathered. The institutions were located in Alabama (Fort Payne), Georgia (Douglasville, Vidalia), Illinois (Algonquin), Indiana (Zionsville), Michigan (Holland), Minnesota (Roseville, Faribault), Missouri (Hannibal), New York (Smithtown), North Carolina (Fayetteville, Hickory), Pennsylvania (Reading, Bethlehem), South Carolina (Anderson, Columbia), and Texas (Austin, Bastrop, Beaumont, Granbury, Hallettsville, Houston, La Grange, Lake Jackson, New Braunfels, Portland, Rosenberg, Spring Branch). The patient characteristics and treatment parameters for these lesions were extracted manually and accessed electronically from written and electronic medical records (EMRs) for all institutions. Lesions included within the data extraction from each institution across the United States were since the initial implementation of the IGSRT program. Exclusion criteria included treatment courses lacking adequate documentation or patients abandoning treatment due to death, noncompliance, or a medical event. Additional data from the EMR, including race, ethnicity, past medical history, medications, follow-up dates, and mortality status/expiratory dates were “data scraped” with algorithmic programming conducted by Sympto Health, Inc.

### 2.3. Ethics

The WCG Institutional Review Board (IRB) waived ethical approval for this work. The dataset was de-identified prior to analysis and all data personnel adhered to the Health Insurance Portability and Accountability Act (HIPAA) and ethical standards to protect patient information.

### 2.4. Statistical Analysis

Freedom from recurrence was estimated using the Kaplan–Meier method. Groups were compared with respect to freedom from recurrence using the log-rank test. All analyses were completed with the intention-to-treat cohort. This cohort was comprised of all patients with NMSC treated with IGSRT, including patients who did not complete a full course of treatment.

## 3. Results

### 3.1. Patient and Tumor Characteristics

A total of 20,069 lesions were included in this retrospective cohort study. A side-by-side comparison of our data (American EMR data from 2016 to 2023) with previous data published by Katalinic et al. (German registry data from 1998 to 2001, excluding recurrences and in situ cases of NMSC), and Duarte et al. (Portuguese registry data from 2011 to 2015) is presented in Table 1 [1,30].

A study by Leiter et al. published in 2014 provides an overview of NMSC epidemiology consistent with our findings as well as those of Katalinic et al. [2] and Duarte et al. [30], reporting that BCCs occur four times as frequently as SCCs and that NMSCs are more common in male versus female patients.

Patient and disease characteristics of the present study are further summarized and stratified by age group in Table 2. Of the 20,069 lesions included, 3158 lesions were in patients aged < 65 years and 16,911 lesions were in patients aged ≥ 65 years. Most lesions in our study were stage 0 (23.3%) or stage 1 (59.6%).

### 3.2. Freedom from Recurrence Rates at Two, Four, and Six Years

Two-year, four-year, and six-year freedom from recurrence rates by age, sex, and stage are presented in Table 3. As shown, the overall 2-year freedom from recurrence rate in the total sample was 99.68%, with freedom from recurrence in 99.71% of younger patients and 99.67% in older patients. Similarly, the overall 4-year freedom from recurrence rate was 99.54%, with freedom from recurrence in 99.71% of younger patients and 99.51% of older patients. Finally, the overall 6-year freedom from recurrence rate was 99.54%, with freedom from recurrence in 99.71% of younger patients and 99.51% of older patients.

### 3.3. Two-Year, Four-Year, and Six-Year Freedom from Recurrence Rates by Age, Sex, and Stage

**Figure 1:**
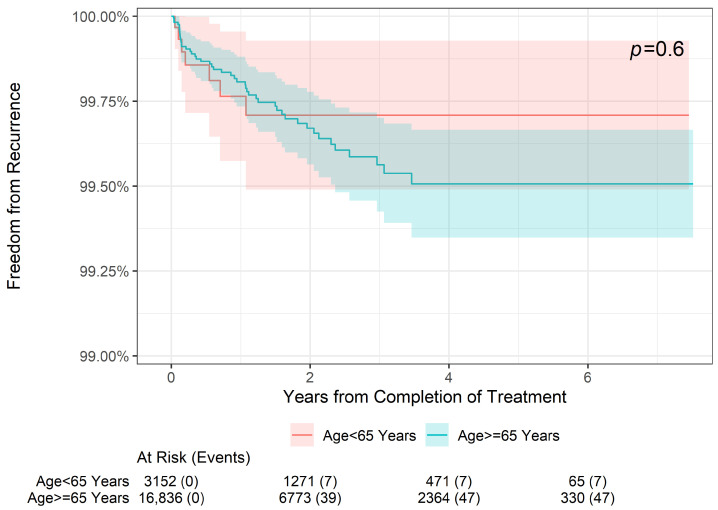
Two-year, four-year, and six-year freedom from recurrence over time of NMSC treated with IGSRT by patient age.

**Figure 2:**
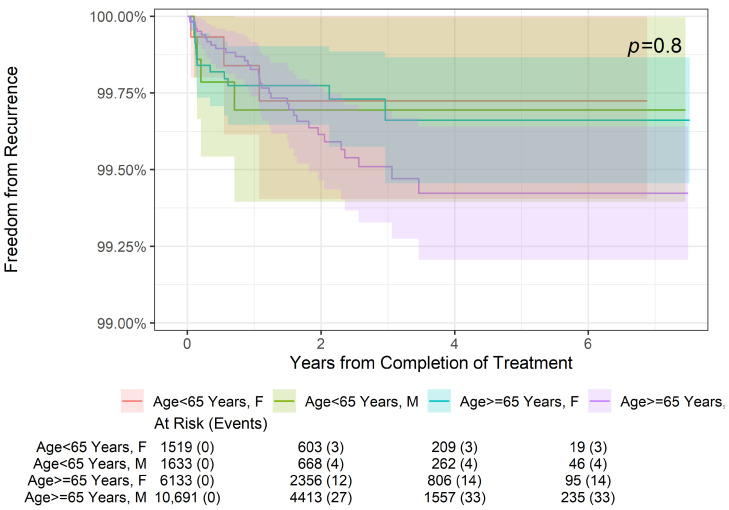
Two-year, four-year, and six-year freedom from recurrence over time of NMSC treated with IGSRT by patient age and sex.

**Figure 3:**
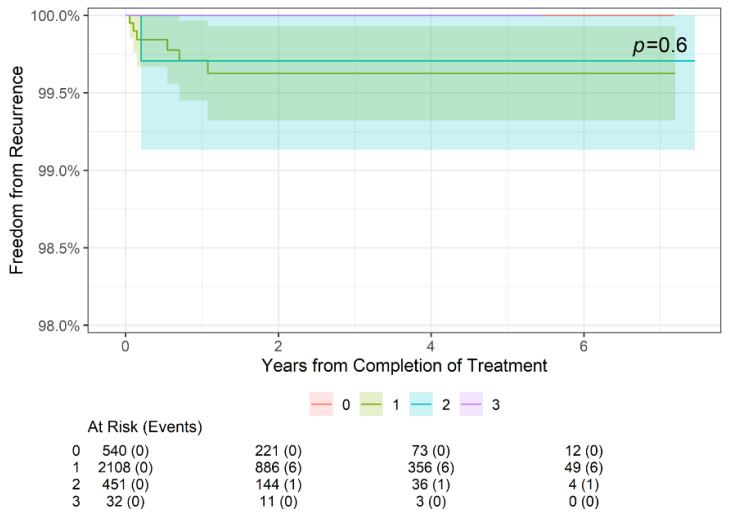
Two-year, four-year, and six-year freedom from recurrence over time of NMSC treated with IGSRT by stage among younger (age < 65 years) patients. AJCC 8th edition staging used.

**Figure 4:**
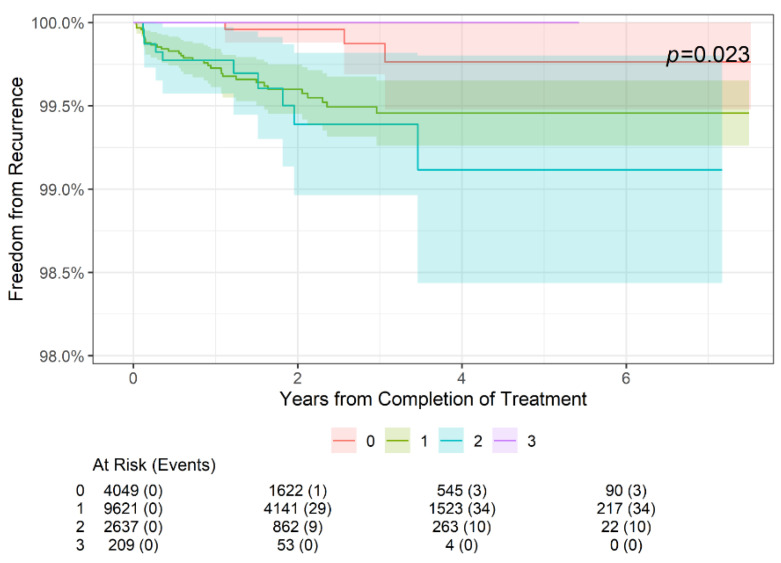
Two-year, four-year, and six-year freedom from recurrence over time of NMSC treated with IGSRT by stage among older (age ≥ 65 years) patients. AJCC 8th edition staging used.

As shown in Figure 1, there was no statistically significant difference in freedom from recurrence at 2 years, 4 years, or 6 years when stratifying patients by age (<65 or ≥65 years old, *p* = 0.6). Further stratification by sex (Figure 2) also did not reveal a significant difference in freedom from recurrence (*p* = 0.8) at 2, 4, or 6 years. Finally, while there was no significant difference in freedom from recurrence when stratified by the American Joint Committee on Cancer (AJCC) 8th edition stage among younger patients aged < 65 years (*p* = 0.6) (Figure 3), older patients (aged ≥ 65 years) did demonstrate a difference in freedom from recurrence when stratified by stage (*p* = 0.023) at 2, 4, and 6 years (Figure 4).

## 4. Discussion

This large retrospective cohort study found that 2-year, 4-year, and 6-year freedom from recurrence following the treatment of NMSC by IGSRT did not vary by patient age (<65 years or ≥65 years) even when stratifying by sex. This lack of difference in recurrence rates was consistent across stages in younger patients, while older patients did demonstrate a significant difference in recurrence when stratified by stage with slightly poorer outcomes seen in patients with stage 2 NMSC. These findings demonstrate that IGSRT is a viable therapeutic option for patients with NMSC regardless of patient age, sex, or stage, and bolster previous findings that IGSRT demonstrates excellent local tumor control and absolute lesion control [26] and superior recurrence rates in this cohort relative to traditional SRT and historical rates of MMS [31].

Notably, most of the lesions included in this cohort were located in the head or neck. This means many lesions were within the H-zone, which is an area of the midface that includes the ears, eyes, and nose. The H-zone is also the site of embryologic fusion, which is associated with the development of more aggressive NMSCs [32,33], and is a cosmetically sensitive area. Complete surgical removal of NMSC clinical target volume (CTV; the gross tumor volume plus margin for subclinical disease spread) in this region therefore poses the risk of facial disfigurement [33]. IGSRT offers a cosmetically favorable treatment option for NMSCs in the H-zone as patients heal without scarring or the need for reconstructive surgery.

The ultrasound component of IGSRT optimizes the assessment of CTV compared to clinical assessment with the naked eye, as evidenced by improved recurrence rates in patients treated with IGSRT versus those treated with SRT. Another technology that may improve CTV assessment is dermoscopy. Dermoscopy is a non-invasive magnification tool that enhances the clinician’s ability to visualize the lateral margins of skin cancers, and therefore may help refine radiotherapy planning [34,35]. In the future, integrating dermoscopy with ultrasound image guidance may further improve upon oncologic outcomes of individuals receiving IGSRT for NMSC.

Superficial interventional radiotherapy (IRT, also called brachytherapy) is another treatment for NMSC. Superficial IRT utilizes surface applicators to deliver radiation to NMSCs and has been reported to have excellent functional and cosmetic outcomes as well as lower treatment costs because it can be performed on an outpatient basis [36]. Furthermore, a novel IRT technique involving the adjustment of catheter-to-skin distance allows for better dose modulation in the treatment of NMSC [37].

The costs of treating NMSC have increased significantly, which represents a substantial burden for patients and healthcare systems and can be an important factor when weighing treatment options [38]. The total cost of MMS care varies by tumor location, stage, tissue blocks, and reconstruction required. As an example, in 2023, for lesions on the legs, arms, or trunk, Medicare reimbursed USD 646.10 for the first non-facility tissue block, USD 401.09 for blocks 2–5, and USD 78.56 per additional block per one lesion [38]. Non-facility reimbursement for lesions on the head, neck, feet, hands, or genitalia or lesions with bone, cartilage, major nerve or vessel, muscle, or tendon involvement was USD 687.63 for the first tissue block, USD 419.08 for blocks 2–5, and USD 78.56 per additional block [38]. On average, 1.7 stages are required for tumor-free margins with MMS [39]. The cost of a 2-stage lesion removal with MMS would therefore be USD1047.19 for a lesion on the arm, trunk, or leg and USD 1106.71 for a lesion in a more challenging area assuming no further intervention or revisions required [40]. Repair costs can range from USD 95.90 for a simple repair of the scalp, axillae, neck, external genitalia, extremities, or trunk to USD 505.60 for more complex repairs such as those required on the ears, nose, or eyelids, excluding the cost of flaps or grafts [40]. These cost projections do not include variations in additional reconstructive surgeries, complications, and recurrences. MMS could therefore have a greater cost range as costs could be less predictable due to the potential for significantly more expensive treatment, reconstruction, and complications, posing potential financial risks for the payer. IGSRT has a smaller potential cost range as costs can be more predictable. Due to the nonsurgical nature of IGSRT, there is no reconstruction as well as limited complications, and up to three lesions are treated simultaneously, comparatively reducing the financial risks for the payer. Other qualitative considerations (e.g., long wait times for MMS, permanent scarring of cosmetically sensitive areas) could potentially increase costs for payers or patients due to higher treatment costs to treat worsening lesions or the costs to treat the negative psychological effects of scars. Further narrowing the total cost of care gap between MMS and IGSRT is recommended.

The most significant limitation of this study is its retrospective design; randomized controlled trials (RCTs) would improve the quality of the evidence available regarding the effects of individual patient and disease characteristics on the effects of IGSRT on freedom from recurrence in patients with NMSC. However, retrospective cohort studies are commonly used to evaluate the efficacy of oncology treatments due to the ethical implications of randomizing patients who require urgent, life-saving treatment. Additionally, while the treatment of patients included in this study was based on TDF calculation tables, these have since been replaced by biologically effective dose (BED) calculations for dose fractionation schedules.

Further retrospective analyses by other patient and disease characteristics, such as histology, tumor location, socioeconomic status, and comorbidity are greatly needed to gather insights into the potential effects of these characteristics on recurrence in patients with NMSC following treatment with IGSRT. Additionally, a higher genomic-adjusted radiation dose (GARD) has been associated with improved outcomes and has been used to independently predict clinical outcomes, including overall survival and time to first recurrence, in patients with a variety of cancers treated with radiation, including NMSC [40,41]. Investigation into personalized radiation regimens based on GARD may therefore facilitate better patient selection and more individualized radiation treatment plans, including plans for treatment with IGSRT.

## 5. Conclusions

This is the first large retrospective cohort study of patients treated with IGSRT for NMSC to evaluate and compare freedom from recurrence by patient age, sex, and stage. Overall, this study found that 2- and 5-year freedom from recurrence rates do not significantly vary between older (age ≥ 65 years) and younger (age < 65 years) patients, nor by sex or stage among the two age groups. In combination with previous cohort studies indicating superiority of IGSRT over SRT, IGSRT demonstrates a good safety profile with predictable outcomes. This suggests that IGSRT is an excellent first-line treatment alternative for patients diagnosed with early-stage NMSCs, regardless of their age, sex, risk level, or stage (0-II).

## Figures and Tables

**Table 1:** Comparison of current study patient characteristics with those of previously published registry studies [1,30].

Patient Characteristic	Present Study (n = 20,069)	Katalinic et al. 2003 [1] (n = 12,956)	Duarte et al. 2018 [31] (n = 72,602)
*Age, median (IQR)*	74.9 (68.2, 81.7)	69.3 (13.1) ^a^	76 (67, 83)
*Sex* Female Male Missing			
	7680 (38.3)	6471 (49.9)	35,267 (48.6)
	12,377 (61.7)	6485 (50.1)	37,335 (51.4)
	12	NA	NA
*Tumor Location*			
Head/neck	12,787 (63.7)	8363 (64.5)	
Ear	1700 (8.5)	475 (3.7)	5104 (7.0)
Face	NA	6014 (46.4)	46,392 (67.0)
Scalp	1297 (6.5)	1000 (7.7)	NA
Forehead	1816 (9.0)	NA	NA
Temple	610 (3.0)	NA	NA
Orbit/Eyelid	121 (0.6)	587 (4.5)	4495 (6.2)
Nose	3467 (17.3)	NA	NA
Cheek	2968 (14.8)	NA	NA
Mucosal lip	51 (0.3)	287 (2.2)	2434 (3.4)
Other face locations	NA	NA	39,463 (54.4)
Chin/Mandible	151 (0.8)	NA	NA
Neck	766 (3.8)	NA	NA
Scalp and neck	NA	1000 (7.7)	6825 (9.4)
Extremities	4142 (20.6)	3179 (24.5)	
Shoulder	468 (2.3)	1058 (7.7)	NA
Upper limb	NA	NA	3502 (4.8)
Trunk	819 (4.1)	2121 (16.4)	6295 (8.7)
Chest	531 (2.6)	NA	NA
Back	793 (4.0)	NA	NA
Lower limb	NA	652 (5.0)	3619 (49.2)
Overlapping anatomical sites	NA	7 (0.1)	NA
Other specified sites	NA	NA	594 (0.8)
Not specified	NA	756 (5.8)	3325 (4.6)
*Histology* BCC SCC SCCIS			
	9928 (49.5)	10,649 (82.2)	28,691 (72.9) ^b^
	5294 (26.4)	2162 (16.7)	10,103 (25.7) ^b^
	4648 (23.2)	NA	NA
≥2 NMSCs	199 (1.0)	NA	NA
Not specified	NA	49 (0.4)	1036 (2.6) ^b^
Others	NA	96 (0.7)	NA

^a^ Presented as mean (SD). ^b^ Histology data are from 39,830 lesions occurring between 2013 and 2015. All data presented as n (%) unless otherwise specified. Abbreviations: BCC, basal cell carcinoma; IQR, interquartile range; n, number of lesions; NA, not available; SCC, squamous cell carcinoma; SCCIS, squamous cell carcinoma in situ; SD, standard deviation.

**Table 2:** Patient and disease characteristics by age.

Patient Characteristic	Patients Aged < 65 Years (*n* = 3158)	Patients Aged ≥ 65 Years (*n* = 16,911)	All Patients (*n* = 20,069)
Age, years, median (IQR)	59.4 (54.5, 62.6)	77.0 (71.5, 82.8)	74.9 (68.2, 81.7)
*Sex* Female Male Missing			
	1522 (48.2)	6158 (36.4)	7680 (38.3)
	1636 (51.8)	10,741 (63.6)	12,377 (61.7)
	0	12	12
*Tumor Location*			
Head/neck	2029 (64.2)	10,758 (63.6)	12,787 (63.7)
Ear	235 (7.4)	1465 (8.7)	1700 (8.5)
Scalp	103 (3.3)	1194 (7.1)	1297 (6.5)
Forehead	295 (9.3)	1521 (9.0)	1816 (9.0)
Temple	83 (2.6)	527 (3.1)	610 (3.0)
Orbit/Eyelid	31 (1.0)	90 (0.5)	121 (0.6)
Nose	681 (21.6)	2786 (16.5)	3467 (17.3)
Cheek	424 (13.4)	2544 (15.0)	2968 (14.8)
Mucosal lip	13 (0.4)	38 (0.2)	51 (0.3)
Chin/Mandible	20 (0.6)	131 (0.8)	151 (0.8)
Neck	118 (3.7)	648 (3.8)	766 (3.8)
Extremities	544 (17.2)	3598 (21.3)	4142 (20.6)
Shoulder	97 (3.1)	371 (2.2)	468 (2.3)
Trunk	205 (6.5)	614 (3.6)	819 (4.1)
Chest	125 (4.0)	406 (2.4)	531 (2.6)
Back	170 (5.4)	623 (3.7)	793 (4.0)
*Stage* 0 1 2 3 Missing			
	541 (17.2)	4091 (24.5)	4632 (23.3)
	2113 (67.4)	9725 (58.2)	11,838 (59.6)
	451 (14.4)	2678 (16.0)	3129 (15.8)
	32 (1.0)	220 (1.3)	252 (1.3)
	21	197	218
*Histology* BCC SCC SCCIS			
	2045 (64.8)	7883 (46.6)	9928 (49.5)
	549 (17.4)	4745 (28.1)	5294 (26.4)
	546 (17.3)	4102 (24.3)	4648 (23.2)
≥2 NMSCs	18 (0.6)	181 (1.11)	199 (1.0)
Lesion size, cm, median (IQR) ^a^	1.0 (0.9, 1.6)	1.0 (0.7, 1.5)	1.0 (0.9, 1.6)
TDF, median (IQR) ^b^	96.0 (93.0, 98.0)	96.0 (93.0, 98.0)	96.0 (93.0, 98.0)
*Energy* ^c^			
100 kV	481 (15.2)	2845 (16.8)	3326 (16.6)
70 kV	1716 (54.4)	9447 (55.9)	11,163 (55.6)
50 kV	925 (29.3)	4460 (26.4)	5385 (26.8)
Other	34 (1.1)	156 (0.9)	190 (0.9)

^a^ Size data missing for 355 lesions (40 in patients < 65 years, 315 in patients ≥ 65 years). ^b^ TDF data missing for 264 lesions (53 in patients < 65 years, 211 in patients ≥ 65 years). ^c^ Energy data missing for 5 lesions (2 in patients < 65 years, 3 in patients ≥ 65 years). All data presented as n (%). Abbreviations: BCC, basal cell carcinoma; n, number of lesions; SCC, squamous cell carcinoma; SCCIS, squamous cell carcinoma in situ; TDF, time–dose–fractionation.

**Table 3:** Two-year, four-year, and six-year freedom from recurrence rates by patient age, sex, and stage.

Patient Characteristic	2-Year Freedom from Recurrence	4-Year Freedom from Recurrence	6-Year Freedom from Recurrence
*Age*			
<65 Years	1271 (99.71)	471 (99.71)	65 (99.71)
≥65 Years	6773 (99.67)	2364 (99.51)	330 (99.51)
Overall	8044 (99.68)	2835 (99.54)	395 (99.54)
*Sex*			
Female	2959 (99.76)	1015 (99.67)	114 (99.67)
Male	5081 (99.63)	1819 (99.46)	281 (99.46)
*Stage*			
0	1851 (99.96)	618 (99.79)	102 (99.79)
1	5047 (99.61)	1885 (99.49)	266 (99.49)
2	1009 (99.44)	302 (99.21)	26 (99.21)
3	63 (100.00)	7 (100.00)	NA

All data presented as n (%). Abbreviations: NA, not available.

## Data Availability

The data presented in this study are available on request from the corresponding author.
